# Supplementary material for: Diet order affects energy balance in randomized crossover feeding studies that vary in macronutrients but not ultra-processing
Source: medRxiv. 2023 Oct 4:2023.10.03.23296501. Preprint. [Version 1] doi: 10.1101/2023.10.03.23296501 (PMC10659501; doi:10.1101/2023.10.03.23296501)
Supplement: Supplement 1 [file NIHPP2023.10.03.23296501v1-supplement-1.pdf]

medRxiv preprint doi: https://doi.org/10.1101/2023.10.03.23296501; this version posted October 4, 2023. The copyright holder for this preprint (which was not certified by peer review) is the author/funder, who has granted medRxiv a license to display the preprint in perpetuity.
This article is a US Government work. It is not subject to copyright under 17 USC 105 and is also made available for use under a CC0 license.

SUPPLEMENTAL MATERIALS

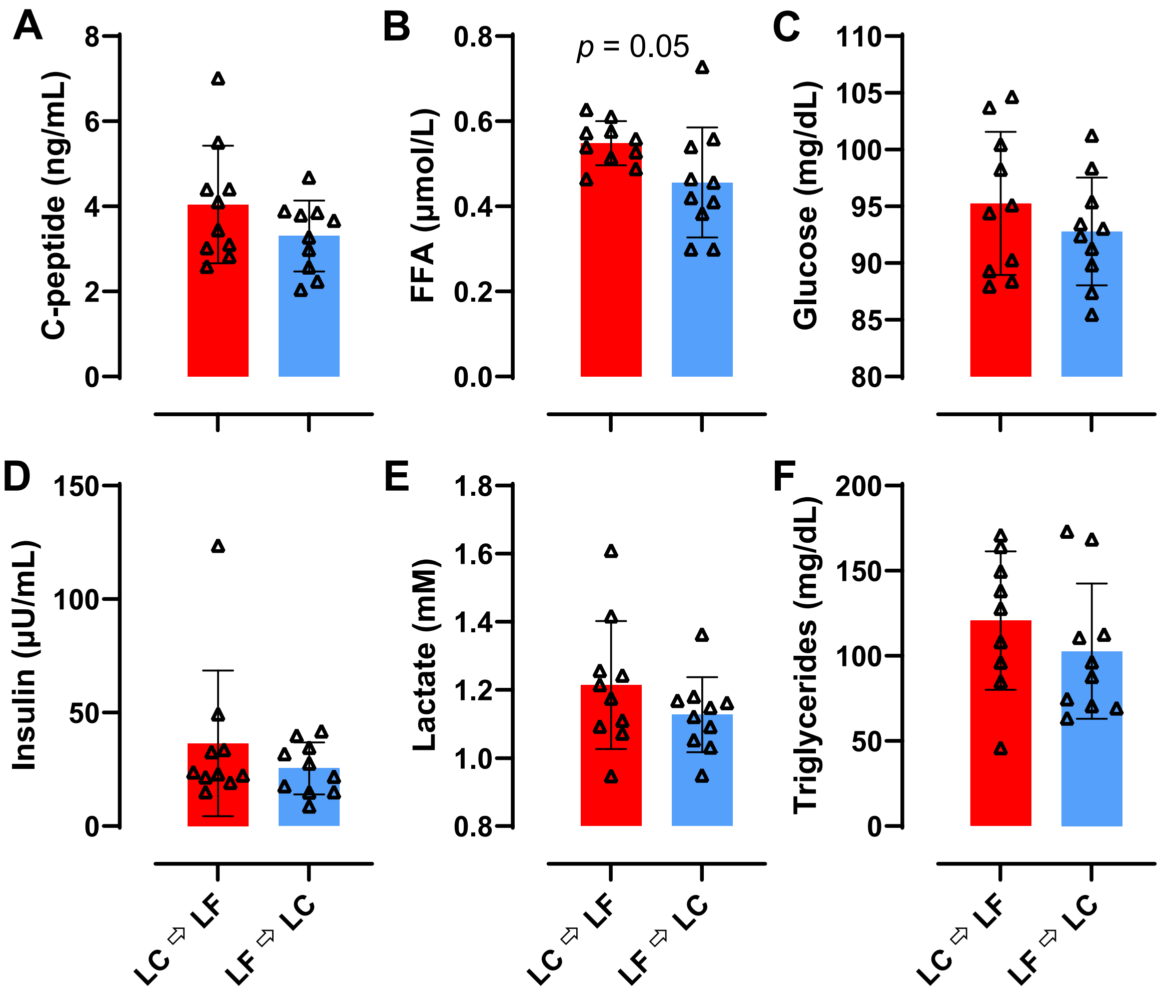

Supplemental Data Figure 1A-F. Diet Order Differences in Postprandial Metabolites During LC vs. LF. Red indicates LC → LF, blue indicates LF → LC. Data were collected during mixed meal tests at 0, 10, 20, 30, 60, 90, 120, 180, 240, 300, and 360 minutes post-meal. Data are total area under the curve (tAUC) and reported as mean ± SEM. *p*-values represent the results of unpaired t-tests.
A) C-peptide tAUC during a mixed meal test.
B) FFA tAUC during a mixed meal test.
C) Glucose tAUC during a mixed meal test.
D) Insulin tAUC during a mixed meal test.
E) Lactate tAUC during a mixed meal test.
F) Triglycerides tAUC during a mixed meal test.

medRxiv preprint doi: https://doi.org/10.1101/2023.10.03.23296501; this version posted October 4, 2023. The copyright holder for this preprint (which was not certified by peer review) is the author/funder, who has granted medRxiv a license to display the preprint in perpetuity. This article is a US Government work. It is not subject to copyright under 17 USC 105 and is also made available for use under a CC0 license.

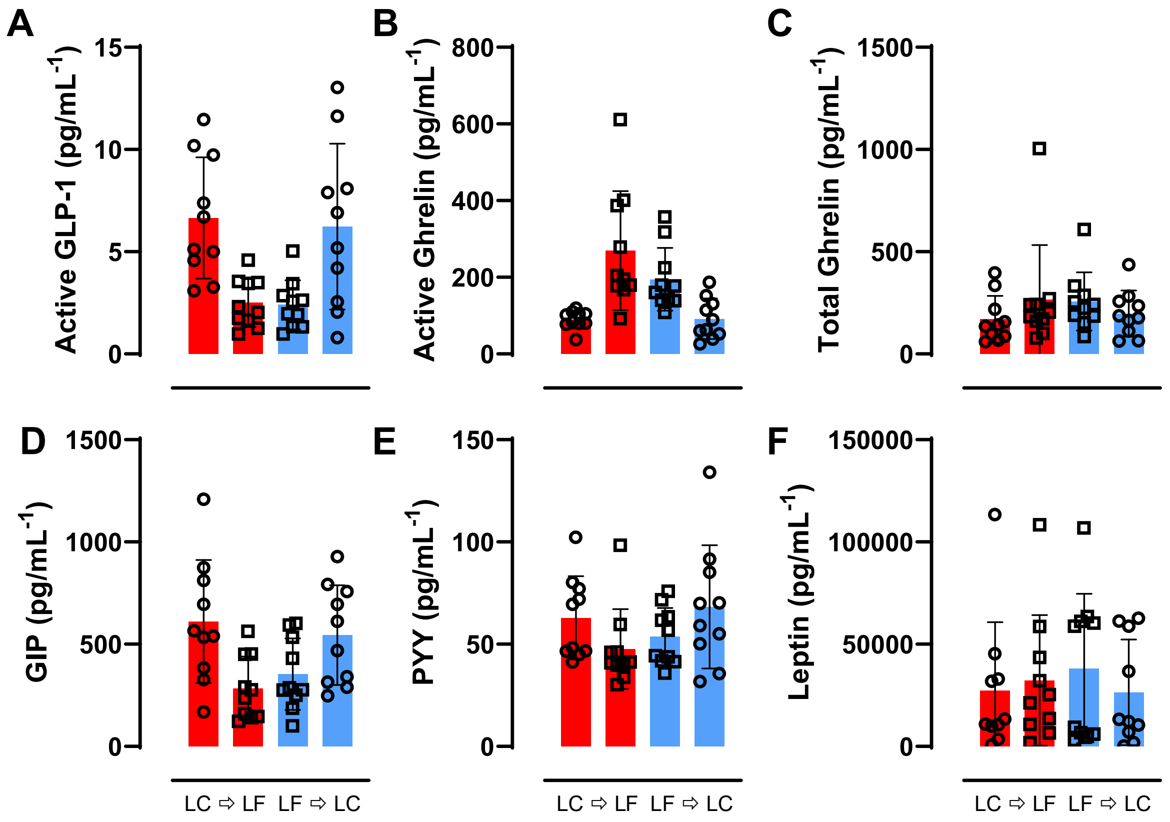

Supplemental Data Figure 2A-F. Diet Order Differences in Gut-derived Hormones During a Meal Test During LC vs. LF. Circles represent the LC diet, squares represent the LF diet. Red indicates LC → LF, blue indicates LF → LC. Data were collected during mixed meal tests at 0, 10, 20, 30, 60, 90, 120, 180, 240, 300, and 360 minutes post-meal. Data are total area under the curve (tAUC) and reported as mean ± SEM. *p*-values represent the results of unpaired t-tests.

A) Active GLP-1 tAUC during a mixed meal test.
B) Active ghrelin tAUC during a mixed meal test.
C) Total ghrelin tAUC during a mixed meal test.
D) GIP tAUC during a mixed meal test.
E) PYY tAUC during a mixed meal test.
F) Leptin tAUC during a mixed meal test.

medRxiv preprint doi: https://doi.org/10.1101/2023.10.03.23296501; this version posted October 4, 2023. The copyright holder for this preprint (which was not certified by peer review) is the author/funder, who has granted medRxiv a license to display the preprint in perpetuity. This article is a US Government work. It is not subject to copyright under 17 USC 105 and is also made available for use under a CC0 license.

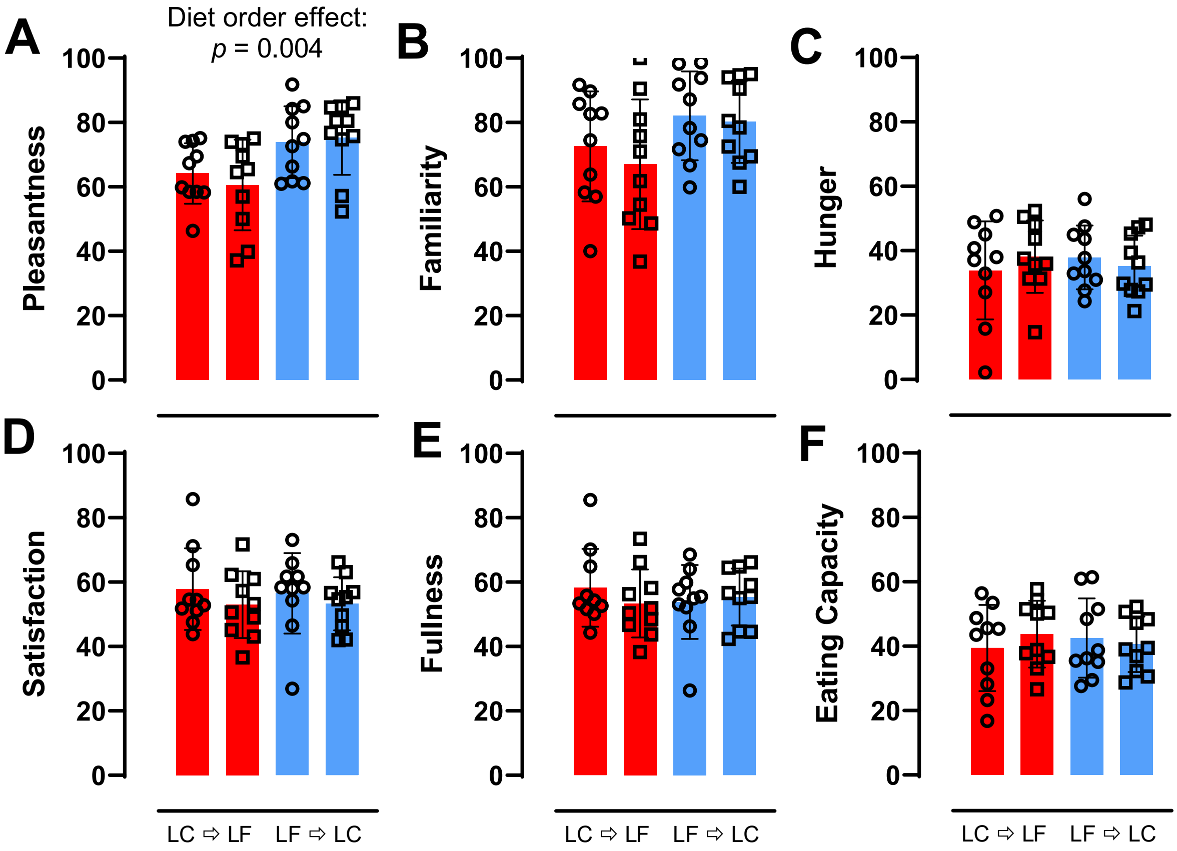

Supplemental Data Figure 3A-F. Diet Order Differences in Subjective Measures of Appetite During LC vs. LF. Circles represent the LC diet, squares represent the LF diet. Red indicates LC → LF, blue indicates LF → LC. Data are mean ± SEM. *p*-values represent the results of unpaired t-tests.
A) Subjective pleasantness of meals by diet order averaged over the 28-day study period.
B) Subjective familiarity of meals by diet order averaged over the 28-day study period.
C) Subjective hunger by diet order averaged over the 28-day study period.
D) Subjective satisfaction by diet order averaged over the 28-day study period.
E) Subjective fullness by diet order averaged over the 28-day study period.
F) Subjective eating capacity by diet order averaged over the 28-day study period.

medRxiv preprint doi: https://doi.org/10.1101/2023.10.03.23296501; this version posted October 4, 2023. The copyright holder for this preprint (which was not certified by peer review) is the author/funder, who has granted medRxiv a license to display the preprint in perpetuity.
This article is a US Government work. It is not subject to copyright under 17 USC 105 and is also made available for use under a CC0 license.

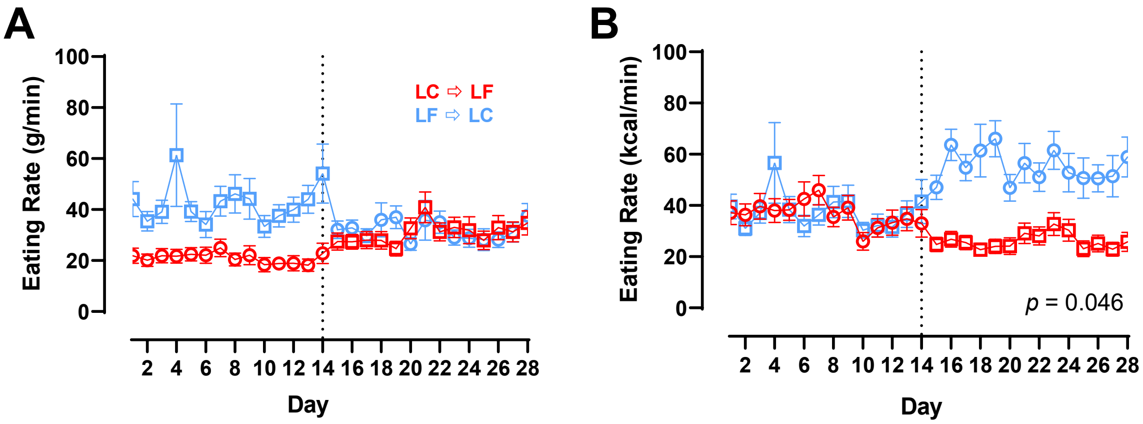

Supplemental Data Figure 4A-B. Eating Rate by Diet Order During LC vs. LF. Circles represent the LC diet, squares represent the LF diet. Red indicates LC → LF, blue indicates LF → LC. Data are mean ± SEM. *p*-values represent the result of unpaired t-tests.

A) Eating rate measured via kcal/min by diet and diet order over 28-day study period.

B) Eating rate measured via g/min by diet and diet order over 28-day study period.

medRxiv preprint doi: https://doi.org/10.1101/2023.10.03.23296501; this version posted October 4, 2023. The copyright holder for this preprint (which was not certified by peer review) is the author/funder, who has granted medRxiv a license to display the preprint in perpetuity.
This article is a US Government work. It is not subject to copyright under 17 USC 105 and is also made available for use under a CC0 license.

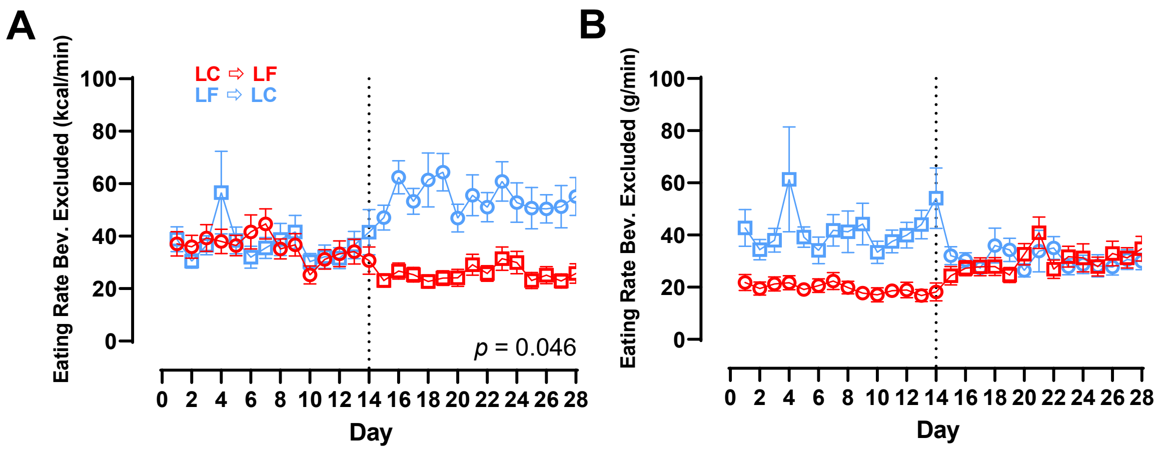

Supplemental Data Figure 5A-B. Eating Rate by Diet Order with Beverages Excluded During LC vs. LF. Circles represent the LC diet, squares represent the LF diet. Red indicates LC → LF, blue indicates LF → LC. Data are mean ± SEM. *p*-values represent the result of unpaired t-tests.

A) Eating rate beverages excluded measured via kcal/min^{-1} by diet and diet order over 28-day study period.

B) Eating rate beverages excluded measured via g/min^{-1} by diet and diet order over 28-day study period.

medRxiv preprint doi: https://doi.org/10.1101/2023.10.03.23296501; this version posted October 4, 2023. The copyright holder for this preprint (which was not certified by peer review) is the author/funder, who has granted medRxiv a license to display the preprint in perpetuity.
This article is a US Government work. It is not subject to copyright under 17 USC 105 and is also made available for use under a CC0 license.

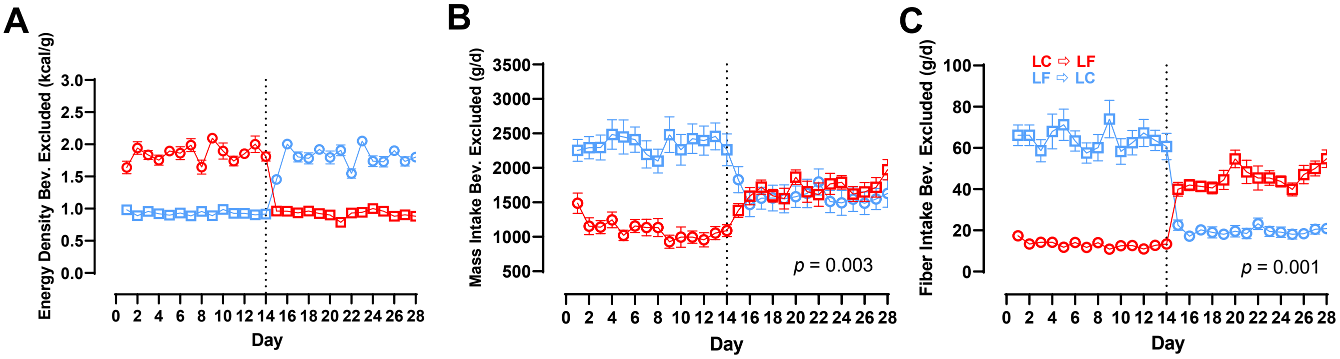

Supplemental Data Figure 6A-C. Energy Density, Mass Intake, and Fiber Intake by Diet Order with Beverages Excluded During LC vs. LF. Circles represent the LC diet, squares represent the LF diet. Red indicates LC → LF, blue indicates LF → LC. Data are mean ± SEM. *p*-values represent the result of unpaired t-tests.

A) Measured energy density beverages excluded by diet and diet order over 28-day study period.

B) Mass intake beverages excluded by diet and diet order over 28-day study period.

C) Fiber intake beverages excluded by diet and diet order over 28-day study period.

medRxiv preprint doi: https://doi.org/10.1101/2023.10.03.23296501; this version posted October 4, 2023. The copyright holder for this preprint (which was not certified by peer review) is the author/funder, who has granted medRxiv a license to display the preprint in perpetuity.
This article is a US Government work. It is not subject to copyright under 17 USC 105 and is also made available for use under a CC0 license.

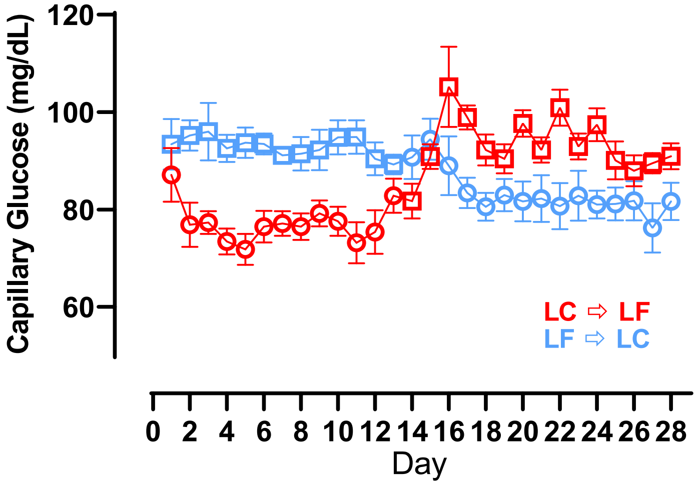

Supplemental Data Figure 7. Capillary Glucose Measured via CGM During LC vs. LF. Circles represent the LC diet, squares represent the LF diet. Red indicates LC → LF, blue indicates LF → LC. Data are mean ± SEM. *p*-values represent the results of unpaired *t*-tests.

medRxiv preprint doi: https://doi.org/10.1101/2023.10.03.23296501; this version posted October 4, 2023. The copyright holder for this preprint (which was not certified by peer review) is the author/funder, who has granted medRxiv a license to display the preprint in perpetuity.
This article is a US Government work. It is not subject to copyright under 17 USC 105 and is also made available for use under a CC0 license.

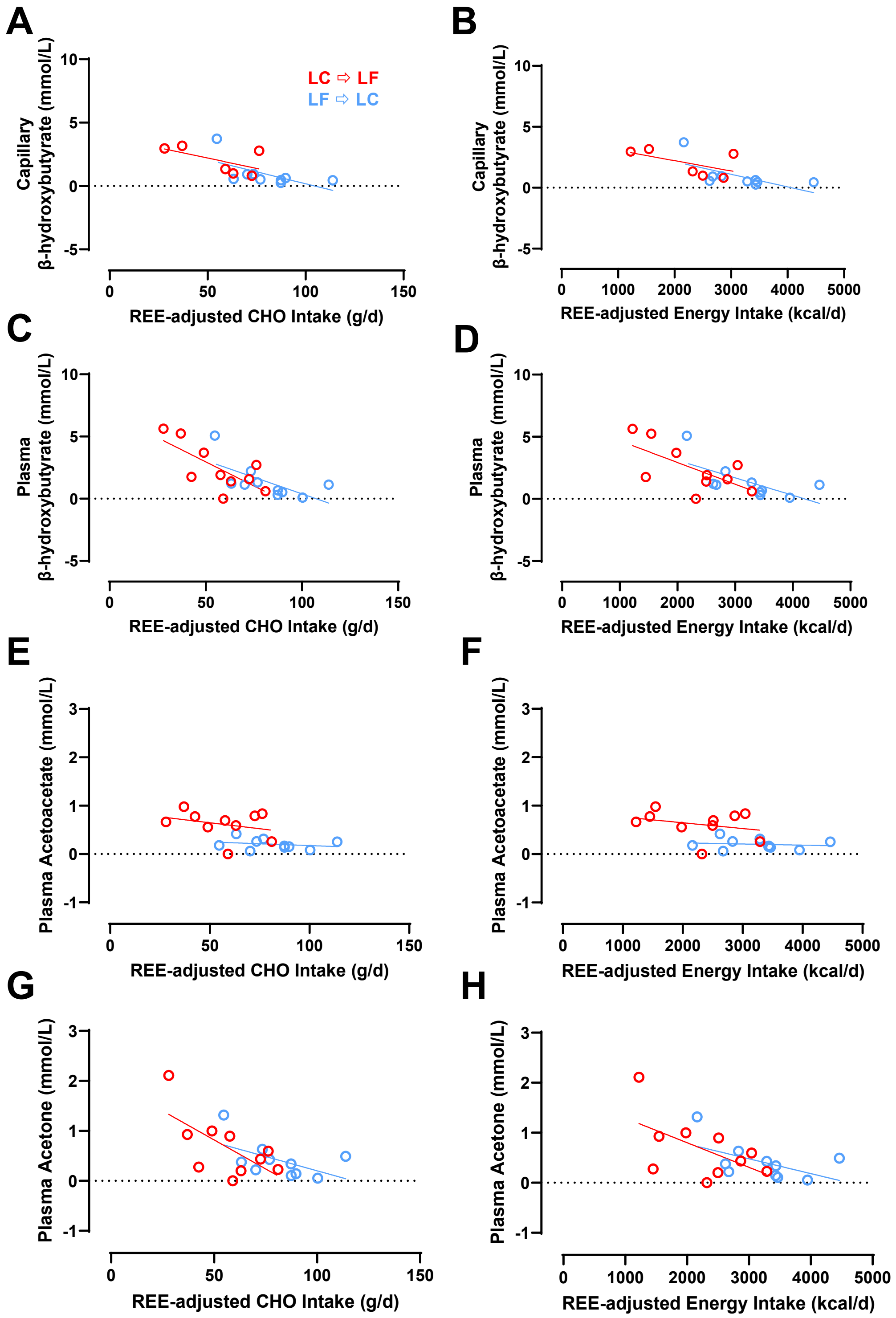

Supplemental Data Figure 8A-H. The Relationship Between Capillary β-hydroxybutyrate, Plasma β-hydroxybutyrate, Plasma Acetoacetate, and Plasma Acetone and Energy and CHO Intake During the LC Diet Only. Circles represent the LC diet. Red indicates LC → LF, blue indicates LF → LC. Data are means.

medRxiv preprint doi: https://doi.org/10.1101/2023.10.03.23296501; this version posted October 4, 2023. The copyright holder for this preprint (which was not certified by peer review) is the author/funder, who has granted medRxiv a license to display the preprint in perpetuity.
This article is a US Government work. It is not subject to copyright under 17 USC 105 and is also made available for use under a CC0 license.

A) Capillary β-hydroxybutyrate and REE-adjusted CHO intake during the 2-week LC diet only.
B) Capillary β-hydroxybutyrate and REE-adjusted energy intake during the 2-week LC diet only.
C) Plasma β-hydroxybutyrate and REE-adjusted CHO intake during the 2-week LC diet only.
D) Plasma β-hydroxybutyrate and REE-adjusted energy intake during the 2-week LC diet only.
E) Plasma Acetoacetate and REE-adjusted CHO intake during the 2-week LC diet only.
F) Plasma Acetoacetate and REE-adjusted energy intake during the 2-week LC diet only.
G) Plasma Acetone and REE-adjusted CHO intake during the 2-week LC diet only.
H) Plasma Acetone and REE-adjusted energy intake during the 2-week LC diet only.

medRxiv preprint doi: https://doi.org/10.1101/2023.10.03.23296501; this version posted October 4, 2023. The copyright holder for this preprint (which was not certified by peer review) is the author/funder, who has granted medRxiv a license to display the preprint in perpetuity. This article is a US Government work. It is not subject to copyright under 17 USC 105 and is also made available for use under a CC0 license.

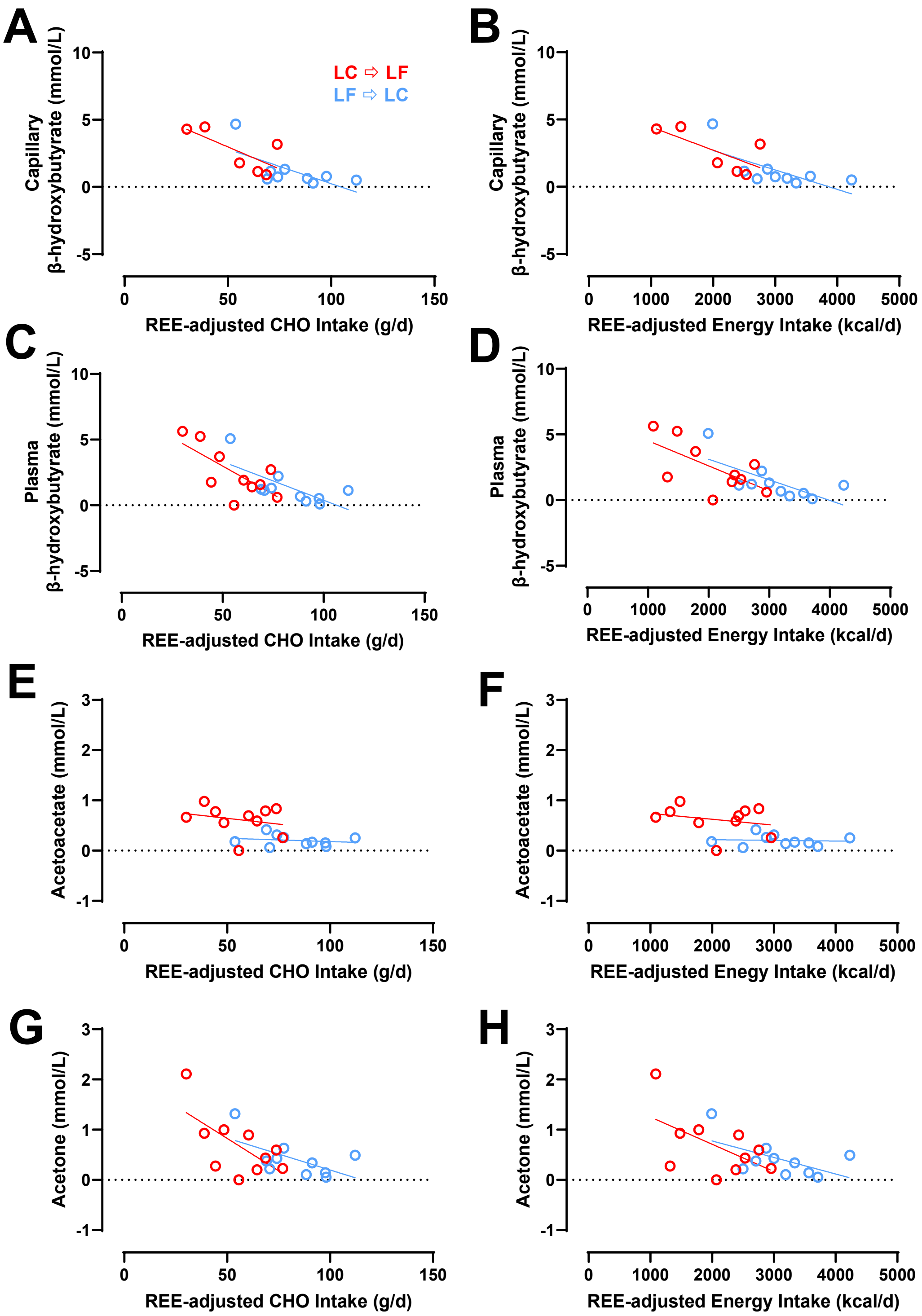

Supplemental Data Figure 9A-H. The Relationship Between Capillary β-hydroxybutyrate, Plasma β-hydroxybutyrate, Plasma Acetoacetate, and Plasma Acetone and Energy and CHO Intake During the Last Week of the LC Diet Only. Circles represent the LC diet. Red indicates LC → LF, blue indicates LF → LC. Data are means.

A) Capillary β-hydroxybutyrate and REE-adjusted CHO intake during the last week of the LC diet only.

B) Capillary β-hydroxybutyrate and REE-adjusted energy intake during the last week of the LC diet only.

medRxiv preprint doi: https://doi.org/10.1101/2023.10.03.23296501; this version posted October 4, 2023. The copyright holder for this preprint (which was not certified by peer review) is the author/funder, who has granted medRxiv a license to display the preprint in perpetuity.
This article is a US Government work. It is not subject to copyright under 17 USC 105 and is also made available for use under a CC0 license.

C) Plasma β-hydroxybutyrate and REE-adjusted CHO intake during the last week of the LC diet only.
D) Plasma β-hydroxybutyrate and REE-adjusted energy intake during the last week of the LC diet only.
E) Plasma Acetoacetate and REE-adjusted CHO intake during the last week of the LC diet only.
F) Plasma Acetoacetate and REE-adjusted energy intake during the last week of the LC diet only.
G) Plasma Acetone and REE-adjusted CHO intake during the last week of the LC diet only.
H) Plasma Acetone and REE-adjusted energy intake during the last week of the LC diet only.

medRxiv preprint doi: https://doi.org/10.1101/2023.10.03.23296501; this version posted October 4, 2023. The copyright holder for this preprint
(which was not certified by peer review) is the author/funder, who has granted medRxiv a license to display the preprint in perpetuity.
This article is a US Government work. It is not subject to copyright under 17 USC 105 and is also made available
for use under a CC0 license.

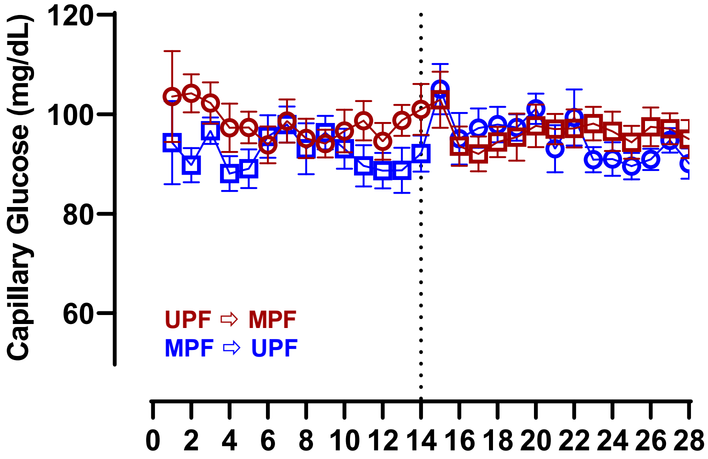

Supplemental Data Figure 10. Capillary Glucose Measured via CGM During UPF vs. MPF. Circles represent the UPF diet, squares represent the MPF diet. Red indicates UPF → MPF, blue indicates MPF → UPF. Data are mean ± SEM.

medRxiv preprint doi: https://doi.org/10.1101/2023.10.03.23296501; this version posted October 4, 2023. The copyright holder for this preprint (which was not certified by peer review) is the author/funder, who has granted medRxiv a license to display the preprint in perpetuity.
This article is a US Government work. It is not subject to copyright under 17 USC 105 and is also made available for use under a CC0 license.

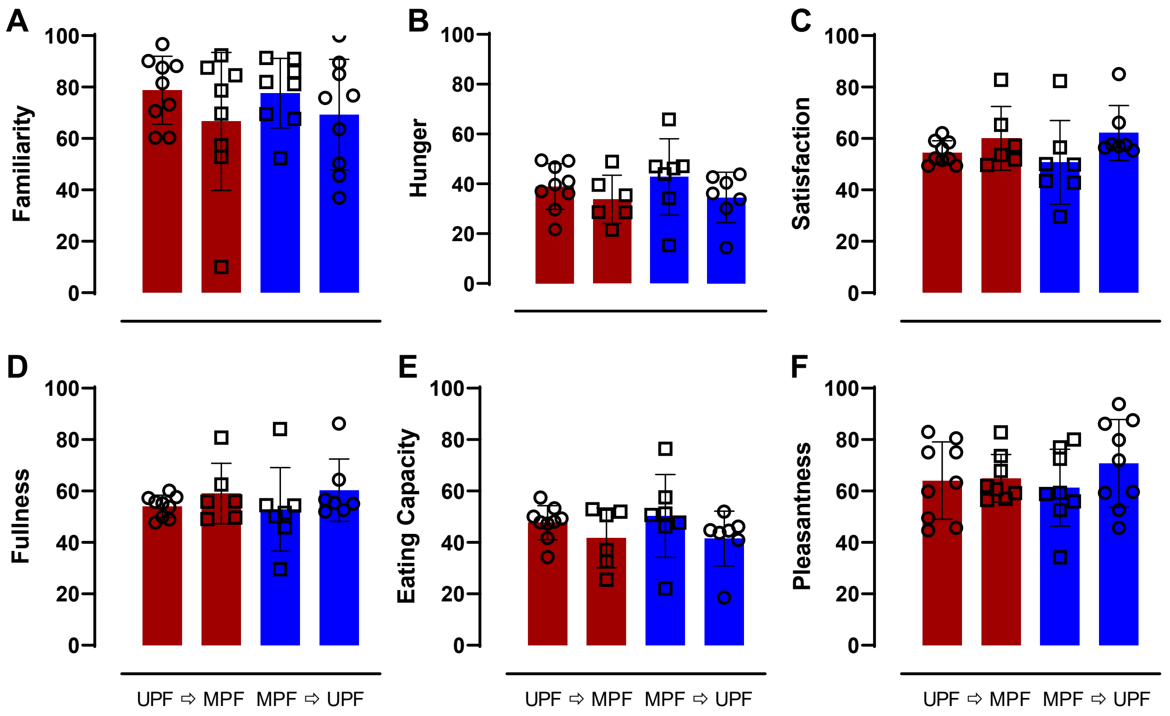

Supplemental Data Figure 11A-F. Diet Order Differences in Subjective Measures of Appetite During UPF vs. MPF. Circles represent the UPF diet, squares represent the MPF diet. Red indicates UPF → MPF, blue indicates MPF → UPF. Data are mean ± SEM.

A) Subjective familiarity of meals by diet order averaged over the 28-day study period. *p*-values represent the results of unpaired t-tests.

B) Subjective hunger by diet order averaged over the 28-day study period.

C) Subjective satisfaction by diet order averaged over the 28-day study period.

D) Subjective fullness by diet order averaged over the 28-day study period.

E) Subjective eating capacity by diet order averaged over the 28-day study period.

F) Subjective pleasantness of meals by diet order averaged over the 28-day study period.

medRxiv preprint doi: https://doi.org/10.1101/2023.10.03.23296501; this version posted October 4, 2023. The copyright holder for this preprint
(which was not certified by peer review) is the author/funder, who has granted medRxiv a license to display the preprint in perpetuity.
This article is a US Government work. It is not subject to copyright under 17 USC 105 and is also made available
for use under a CC0 license.

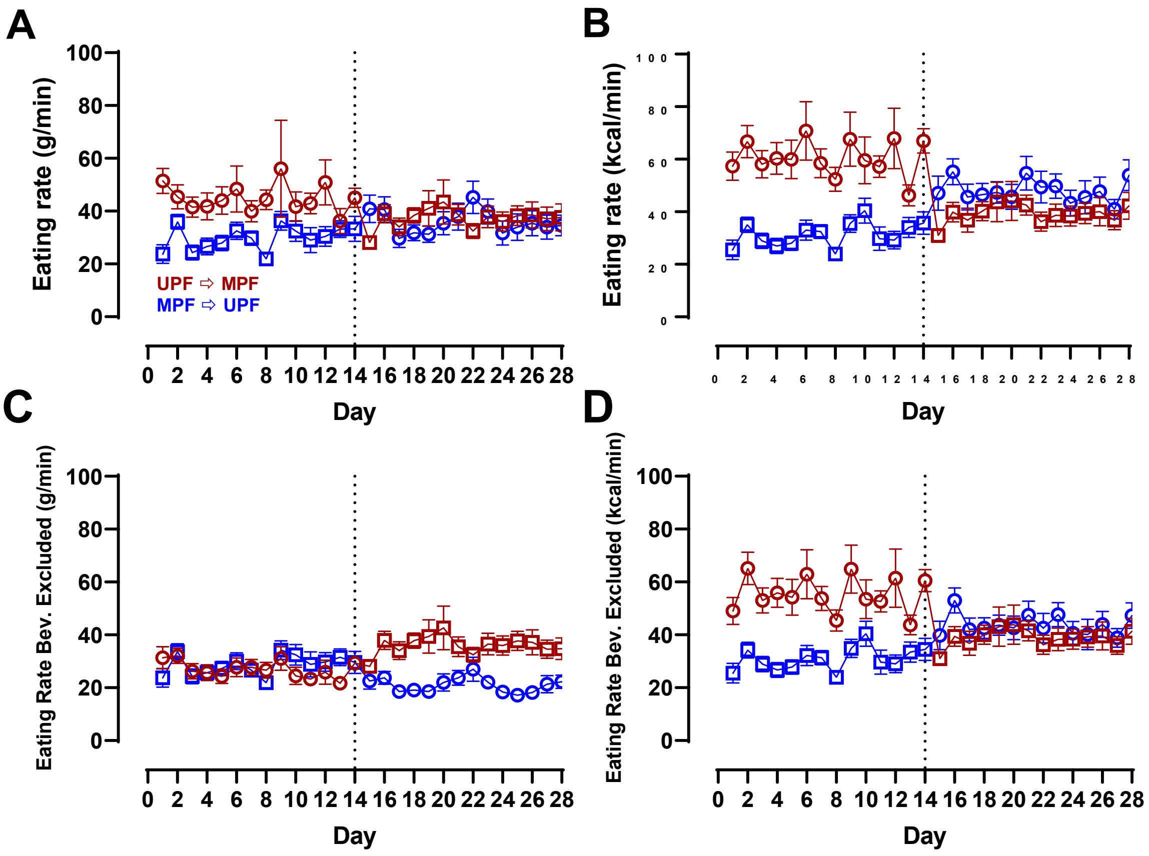

Supplemental Data Figure 12A-D. Eating Rate by Diet Order During UPF vs. MPF. Circles represent the UPF diet, squares represent the MPF diet. Red indicates UPF → MPF, blue indicates MPF → UPF. Data are mean ± SEM.

A) Eating rate measured via g/min by diet and diet order over 28-day study period.

B) Eating rate measured via kcal/min by diet and diet order over 28-day study period.

C) Eating rate measured via g/min by diet and diet order over 28-day study period with beverages excluded.

D) Eating rate measured via kcal/min by diet and diet order over 28-day study period with beverages excluded.
